# Perspectives of Black and White Family Members on Medical Decision Making for ICU Patients

**DOI:** 10.1007/s11606-026-10213-z

**Published:** 2026-01-29

**Authors:** Astha Ray, Kayla N. Thompson, Kimberly S. Johnson, Christopher E. Cox, Martha Lee, Katelyn Dempsey, Deepshikha C. Ashana

**Affiliations:** 1 Trinity College of Arts and Sciences, Duke University, Durham, NC USA; 2 Department of Medicine, Northwestern University, Chicago, IL USA; 3 Department of Medicine, Duke University, Durham, NC USA; 4 Geriatrics Research Education and Clinical Center (GRECC), Durham Veterans Affairs Health Care System, Durham, NC USA; 5 University of North Carolina at Chapel Hill, Chapel Hill, NC USA; 6 Duke-Margolis Center for Health Policy, Duke Univsersity, Durham, NC USA; 7 Department of Population Health Sciences, Duke University, Durham, NC USA

**Keywords:** communication, equity, family, ICU, patient-centered, shared decision making, trust

## Abstract

**Background:**

In intensive care units (ICUs), shared decision making (SDM) between clinicians and families is critical for evaluating complex tradeoffs of life-sustaining treatments and making patient-centered decisions, yet clinicians are less likely to engage in SDM with Black compared to White families.

**Objective:**

To describe how Black compared to White family members experienced medical decision making about life-sustaining treatments for their critically ill loved ones.

**Design:**

This study is a thematic analysis of semi-structured, audio-recorded interviews with primary surrogate decision makers. The codebook included recommended components of SDM and known mechanisms of communication disparities. Analysts were blinded to family member race during coding; then patterns between and within racial groups were analyzed to identify themes.

**Participants:**

Black or White primary surrogate decision makers for patients mechanically ventilated for ≥ 48 h.

**Main Measures:**

Themes describing experiences of medical decision making.

**Key Results:**

In 43 interviews, both Black (*n* = 25, 58%) and White (*n* = 18, 42%) family members were middle aged (median [interquartile range, IQR]: 55 [15] years vs 58 [17] years) and had critically ill loved ones with similar lengths of ICU stay (median [IQR]: 18 [22] days vs 23 [18] days). Black family members disproportionately reported the following: (1) experiencing pressure to make decisions that aligned with the ICU team’s recommendations and timeline, (2) needing to code-switch by modifying their communication and behavior to ensure their advocacy was welcomed rather than perceived as intrusive, (3) being disregarded by the medical team, which negatively impacted partnership towards shared decisions, and (4) trusting clinicians’ competence but not necessarily their intentions for critically ill loved ones.

**Conclusions:**

Compared to White family members, Black family members of critically ill patients experienced unique challenges relevant to SDM. These results may identify promising focus areas for future conceptual models and interventions to improve equity in ICU-based SDM.

**Supplementary Information:**

The online version contains supplementary material available at 10.1007/s11606-026-10213-z.

## INTRODUCTION

Critical care professional societies endorse shared decision making (SDM) as a central component of person-centered critical care.^1,2^ SDM is a collaborative approach to medical decision making that engages clinicians, patients, and families to align medical treatments with patients’ preferences for their medical care.^2^ SDM is uniquely important in intensive care unit (ICU) settings because complex tradeoffs exist between the potential benefits and risks of life-sustaining treatments such as mechanical ventilation.^3,4^

In the United States, research has found that clinicians are less likely to engage in SDM with Black compared to White patients and families.^5–15^ In ICUs, clinicians share less medical information and provide less emotional support for difficult decisions when communicating with Black compared to White families.^16^ Identifying racially disparate features of SDM is a critical step towards developing interventions to promote equitable SDM. However, much of the medical literature relevant to racial differences in SDM has been reported from the perspective of clinicians and may neglect aspects of SDM that families value most.^16,17^ The experiences and priorities of Black family members engaged in decision making on behalf of their critically ill loved ones are not known.

To address this gap, we conducted a qualitative study to describe the experiences of Black family members who made decisions about life-sustaining treatments on behalf of their critically ill loved ones. We also included White family members to distinguish between experiences that were unique to Black family members versus those shared by both Black and White family members.

## METHODS

This qualitative study received approval from the Duke University Health System Institutional Review Board and adhered to the Consolidated Criteria for Reporting Qualitative Research (COREQ) guideline (Supplement 1).^18^

### Setting and Participants

Eligible family members (an inclusive definition comprising family, friends, or other loved ones)^19^ were English-speaking adults > 17 years old who self-identified as Black or White and acted as primary surrogate decision makers for patients mechanically ventilated for ≥ 48 h in 5 medical or surgical ICUs in one suburban-rural health system in the Southeastern United States. In a subset of cases, family members also reported traumatic stress symptoms.^20–22^ Family members meeting eligibility criteria were randomly selected based on coordinator availability and approached by experienced White female research coordinators (M.L., K.D.). Family members were approached either in person or via phone after patients were ventilated for 48 h in the ICU and asked if they wanted to participate in a single interview about their experiences with ICU medical decision making. Sociodemographic data were provided by participants at the time of consent, and an interview was scheduled based on the mutual availability of the participant and interviewer. Interview timing is detailed in Table 1. Family members were compensated $30 for their participation.

**Table 1:** Family Member and Patient Characteristics

Characteristics	Black family members (*n* = 25)	White family members (*n* = 18)
*Interview characteristics*		
Duration in minutes, median (IQR)	51 (16)	55 (15)
Completed during ICU stay, *n* (%)	5 (20)	5 (28)
Completed after ICU stay, *n* (%)	20 (80)	13 (72)
Days after discharge, median (IQR)	15 (15)	19 (17)
*Patient characteristics*		
Age in years, median (IQR)	62 (11)	66 (16)
Sex, *n* (%)		
Female	8 (32)	8 (44)
Male	17 (68)	10 (56)
Race, *n* (%)		
Black	25 (100)	0 (0)
White	0 (0)	18 (100)
Insurance coverage, *n* (%)		
Commercial	7 (28)	7 (39)
Medicaid	4 (16)	0 (0)
Medicare	12 (48)	11 (61)
None	2 (8)	0 (0)
Number of comorbidities, median (IQR)*	4 (2)	4 (2)
Days in ICU, median (IQR)	18 (22)	23 (18)
Alive at hospital discharge, *n* (%)	18 (72)	10 (56)
*Family member characteristics*		
Age, median (IQR), years	55 (15)	58 (17)
Sex, *n* (%)		
Female	21 (84)	14 (78)
Male	4 (16)	4 (22)
Relation to patient, *n* (%)		
Child	9 (36)	5 (28)
Parent	2 (8)	0 (0)
Sibling	3 (12)	1 (6)
Spouse or partner	11 (44)	11 (61)
Other	0 (0)	1 (6)
Religiosity, median (IQR)^a^*	99 (6)	100 (19)
Health literacy, median (IQR)^b^	12 (4)	14 (4)
Social support, *n* (%)^c^		
Definitely true	20 (80)	13 (72)
Probably true	3 (12)	3 (17)
Probably false	0 (0)	1 (6)
Definitely false	2 (8)	1 (6)
Highest level of education, *n* (%)		
Advanced degree	3 (12)	3 (17)
College graduate	9 (36)	10 (56)
Some college	9 (36)	4 (22)
High school graduate	4 (16)	1 (6)
Quality of clinician communication, median (IQR)^d^	10 (2)	10 (2)
Any conflict with clinicians, *n* (%)^e^	10 (40)	5 (28)

^*^Missing data counts: 5 for the number of comorbidities and 1 for religiosity

^a^The question asked, “How important is your faith or spirituality in your daily life on a scale of 0 (not at all) to 100 (very important)?”

^b^Self-reported 3-item instrument with summed scores ranging from 3 to 15 and higher scores corresponding to increasing health literacy.^23^

^c^The question asked, “I feel that I have someone I can turn to for advice about making important decisions (like medical decisions).”

^d^The question asked, “Overall, how would you rate the ICU doctor’s communication with you (0 is the worst you could imagine and 10 is the best you could imagine)?”

^e^The question asked, “How much disagreement, including conflicts and negative feelings, has there been between you and your ICU doctors regarding your loved one’s care (with 0 being none and 10 being the most you could imagine)?” Responses were dichotomized to indicate no conflict (0) or any conflict (> 0)

### Data Collection

An interview guide (Supplement 2) was iteratively developed by D.C.A., an Asian female critical care physician and experienced qualitative investigator, based upon ICU SDM practice guidelines, critical conceptual models of SDM by Peek and Joseph-Williams that emphasize the dual importance of knowledge and power in making shared decisions, and feedback from other investigators (K.S.J., a Black female geriatrics physician, and C.E.C., a White male critical care physician).^1,24–26^ Questions used narrative prompts about experiences of decision making in the ICU and semi-structured prompts about trust, emotional support, and relationship with the ICU team as related to decision making. D.C.A. and research assistants trained in interview methods conducted virtual, audio-recorded interviews via telephone or videoconferencing software. No relationship was established between participants and investigators prior to interviews. Data collection continued until thematic saturation was reached; the team evaluated in real time whether new information was learned in interviews and recruitment was stopped after two successive interviews yielded no new information.^27,28^ Demographic and clinical data were collected through family self-report and the electronic health record.

### Data Analysis

Interview recordings were professionally transcribed and de-identified. A.R. (an Asian female undergraduate student), K.T. (an Asian female medical student), and D.C.A. developed a codebook (Supplement 3) comprising deductive codes based on existing SDM literature and inductive codes based on the transcripts. Two coders (A.R. and K.T.) blinded to family member race conducted concurrent open coding of all transcripts using NVivo 14 and resolved discrepancies via consensus. All transcripts were double coded with discrepancies in code application resolved via group discussion and consensus. We conducted thematic analysis to identify patterns of racial differences in SDM experiences;^29^ after being unblinded to race, A.R., K.T., and D.C.A. used code frequencies and matrix coding to independently identify potential themes. Themes were iteratively revised and finalized after consensus was reached through group discussion.

## RESULTS

Eighty-two family members were approached for interviews, 60 (73%) consented to participate, and 43 (52%) completed interviews. Among 43 interviewed family members, 25 (58%) identified as Black and 18 (42%) as White. Interview duration was similar for Black and White family members (median [IQR]: 51 [16] min vs 55 [15] min). Both Black and White family members were generally middle aged (median [IQR]: 55 [15] years vs 58 [17] years) and female (*n* [%]: 21 [84] vs 14 [78]). Black and White patients spent similar lengths of time in the ICU (median [IQR]: 18 [22] days vs 23 [18] days). Black patients were more commonly alive at hospital discharge compared to White patients (*n* [%]: 18 [72] vs 10 [56]). Table 1 shows family and patient characteristics.^23^

In interviews, family members described making decisions about life-sustaining treatments (e.g., dialysis and mechanical ventilation), surgical procedures, and comfort-oriented care. Both Black and White family members emphasized that the following clinician behaviors facilitated SDM: being transparent about uncertainty and the patient’s expected prognosis and showing personalized care for the patient (e.g., speaking to the patient even while they were sedated and using the patient’s preferred name).

The following components of Black family members’ experiences hindered their engagement in medical decision making: (1) experiencing pressure to make decisions, (2) needing to code-switch by modifying communication and behavior to fit in, (3) being disregarded by the medical team, and (4) perceiving tension between trusting clinicians’ competence versus intentions (Table 2). Themes did not vary within the subset of cases reporting traumatic stress.

**Table 2:** Additional Representative Quotes for each Theme

Theme	Quotes from Black family members	Quotes from White family members
1. Experiencing pressure to make decisions	“What could have been better is not the last minute. Like I know that they said that, that [removing the defibrillator] might be a possibility, but when they know that that’s the only possibility… Kind of inform the family or individual or whatever at that time, instead of a day before… Now all of a sudden his blood pressure is still dropping… ‘we’re gonna have to take the defibrillator [out]’ and what can I say? Because you all know it so I’m gonna have to go along with it. I can’t say no because he already, [patient]’s sick.” (ID 331, 56-year-old female, spouse of 57-year-old patient) "I didn’t think he was ready physically and I didn’t think he was ready mentally but they told me that they had to do it [surgery]… I didn’t like the fact that you know, I had to make these decisions [about surgery] based on what they, what they had said to me. And they only said those things because they need to have a consent. But nothing [no communication] really after." (ID 292, 61-year-old female, spouse of 75-year-old patient)	“All the doctors, several of them, seemed to want to talk with me about end-of-life decisions… But, this is what I ended up telling the team. I will know when it’s time… They stopped asking me after that. I had them explain comfort care, said thank you, and that was the end of that. And when we had the conversation next, it was because I initiated it.” (ID 152, 68-year-old female, spouse of 80-year-old patient) “I think they gave me my time to make the decision [to withdraw ventilation]. I know nobody told me ‘well, you need to take this off right now.’ Yeah, I was, I think I was supported… They were always there and they weren’t like here and gone again, you know… The neurologist came in and he walked over and held my hand and stood with me.” (ID 124, 77-year-old female, spouse of 80-year-old patient)
2. Needing to code-switch by modifying communication and behavior to fit in	“I also want to make sure I’m not in your [the ICU team’s] way. And I hope that I didn’t bother anyone… I had my own concerns of not being that patient’s family member that kind of hovers, but I am very inquisitive.” (ID 133, 41-year-old female, child) “When I talked to the social worker, I told him I wasn’t prepared [for discharge]… Each time I put ‘me’ in or ‘I’, I had to say… I know it’s not about me… I was saying I’m still trying to straighten up his room… I didn’t want anyone to think I was making it about me.” (ID 575, 62-year-old female, sibling of 55-year-old patient)	“I was fighting for my husband’s life… I felt safe telling everybody how I felt all the time. I didn’t bite my tongue for nobody, you know?" (ID 406, 60-year-old female, spouse of 61-year-old patient) “I said, look, I think we’re beginning to see some signs of depression on her part, and before we fall down this path, is there anything we can do? He [doctor] looked, he looked at me, and he said, let’s do this he said, I’ll get 2 nurses and 2 respiratory therapists, and we’ll take her outside.” (ID 109, 66-year-old male, spouse of 67-year-old patient)
3. Being disregarded by the medical team	“Everybody needs to be treated as a person and be made to feel that they are worth something… The doctor that came in and kept looking at his watch every two minutes… It’s like I don’t have time for this… This is the way he made me feel.” (ID 290, 82-year-old female, parent of 65-year-old patient) "They [doctors] came in, they saw him, they [examined] him, and then they was gone… At least let me know or let him [patient] know what’s going on and what it is for.” (ID 87, 52-year-old female, spouse of 56-year-old patient) "[Communication was] kind of like telling me my total at the market… It wasn’t really any regard for… how we felt… Give me a minute to collect myself before you continue since obviously this is hard to hear, but instead of ‘OK, take your time,’ it was more of a ‘Well, I’m going to continue on and hopefully you catch up sort of thing.’” (ID 281, 27-year-old female, child of 50-year-old patient)	“The nurses, in particular, did an excellent job of making sure that we didn’t feel like alone… When the one got engaged, the other one baked her a cake. They brought me cake. They did a really good job of, you know, building kind of a little, a tiny little one room community.” (ID 196, 44-year-old female, spouse of 45-year-old patient) “I mean both teams I feel like would ask my opinion too. Like do I think he’s feeling this way, do I think he’s feeling that way, and I would say what I thought… I do think that they were very open to listening.” (ID 342, 45-year-old female, spouse of 48-year-old patient) “Well, I believe it was all of us [family and doctors making treatment decisions together]… They were very good about coming back and explaining things a couple times to me.” (ID 79, 73-year-old female, spouse of 76-year-old patient)
4. Perceiving tension between trusting clinicians’ competence versus intentions	“The nurse had blocked anybody from coming [into the ICU] because she got into it with my niece… My husband is back there dying and I can’t even be back there…. How can I trust her to take care of my husband when that happened?” (ID 227, 63-year-old female, spouse of 74-year-old patient) “I have a lot of confidence in [Institution]. I was born there and I worked there… So, I have a lot of confidence in [Institution]. I just don’t like the non-communication… The nurse didn’t show up for at least an hour and then she was in a hurry and I thought ICU was [meant to be attentive because] you have your own nurse.” (ID 292, 61-year-old female, spouse of 75-year-old patient)	“Even when things were not in a crisis situation, they were constantly checking in…It makes you feel like they care when they’re intentionally finding out or people are telling them, ‘Hey look, the family’s here,’ and they’re immediately coming to the room to talk to you. And it’s not just a quick ‘Hi, I’m doctor whoever. I’m taking care of your dad. If you have questions, let me know,’ and leaving.” (ID 93, 36-year-old female, child of 72-year-old patient) “I felt like they were very open with the possibilities of what was going to happen and I think that helped build trust in the beginning… It was not all roses. It was [about]… the side effects, the possible complications, so that helped build trust… They did consult among themselves. That’s one of the things I really like is it wasn’t just one doctor making the decision. They would consult with multiple doctors together.” (ID 175, 56-year-old male, spouse of 59-year-old patient)

### Experiencing pressure to make decisions

Some Black family members felt coerced to choose the treatment pathway recommended by the ICU team and to do so quickly. In contrast, White family members did not report pressure or judgment from the ICU team surrounding decision making.

One Black family member felt pressured to withdraw mechanical ventilation, but she only did so when a new attending physician gave her time to consider her faith:

“They [doctors] said… I need to hurry up and take her off the life support… I felt real pressure with them. I told them I wasn’t ready right then … She [the doctor] looked at me like I was crazy.

Then Dr. [redacted name] came… He was so pleasant. He, that’s why I ended up like taking her off the breathing machine… It’s just like my heart was so content then… He was like, just pray about it and he’s like at the end of the day, God has the last word.” (ID 117, 52-year-old daughter of 73-year-old patient)

Another Black family member reported that her ability to make decisions was hindered by unnecessary urgency from the medical team:

“They kept coming at me for me to make decisions [about discontinuing mechanical ventilation]. And then when you ask them, you know, do it have to be made now? They’d be like, no, it don’t have to be made now... They could not drill, try to drill it in you over and over and over… They put you back in a dark place… I had to come home. I had to clear my mind.” (ID 121, 53-year-old wife of 56-year-old patient)

A White family member, however, was never questioned about her family’s decision to pursue a tracheostomy for her father despite his preference for a do-not-resuscitate (DNR) code status. She explained that it is important for clinicians to respect family decisions; otherwise, families may doubt that they made a good decision, even long after the ICU stay:

“I never got that feeling [that clinicians disagreed with the decision to pursue tracheostomy]. In fact, I feel like we got the opposite feeling. Like whatever decision we made was an OK decision to make… And since ultimately, when it comes to my dad, all of that responsibility [of decision making] is on me, it’s nice to feel like nobody’s looking at you for making the wrong choice.” (ID 93, 36-year-old daughter of 72-year-old patient)

### Needing to code-switch by modifying communication and behavior to fit in

Some Black family members censored their natural communication style or behavior to avoid being perceived negatively by the medical team. No White family members reported code-switching to gain the team’s approval.

In one case, a Black family member avoided disagreeing with the team’s management because she feared repercussions for herself and her loved one:

“I try not to create a ruckus because I don’t want them to say, well you just don’t need to come up here no more… I just bite my tongue… For me to do that [question the clinician], it probably wouldn’t have went over too well… I don’t want to think that he [patient] would be slighted for something that I said or done.” (ID 292, 61-year-old wife of 75-year-old patient)

One Black family described medical team actions that reinforced the need for self-censorship. She was not informed when her husband’s condition deteriorated, causing his transfer to the ICU. When she expressed disappointment about the lack of communication, the bedside nurse seemed to avoid further communication with her:

“I talked to the nurse that was there cause they… were the ones that transfer… They brushed it off like, ‘You don’t understand… in the middle of the night… we weren’t wantin’ to disturb your sleep’… When I told her, she left and she didn’t ever come back.” (ID 331, 56-year-old wife of 57-year-old patient)

In contrast, a White family member exercised unrestrained advocacy for her loved one, even if it meant upsetting the team:

“I wanted them to understand I was not okay with the way they were doing that [talking over the patient’s uncovered body]… I didn’t care [how I was perceived]… They told me everyone needed an advocate like me. And they were glad that I was there.” (ID 152, 68-year-old wife of 80-year-old patient)

### Being disregarded by the medical team

Many Black family members shared experiences where they or their critically ill loved ones were devalued or ignored by the medical team. In contrast, most White family members described strong interpersonal relationships with the ICU team.

One Black family member felt traumatized by the lack of empathy shown by the medical team when discussing her mother’s poor prognosis:

“I’m an only child, [my mother] is my best friend… [The doctors] act like… let’s just get rid of this one little patient and we’re going to the next one… They treated me like I was nothing and she wasn’t nothing. Yeah, that’s, that’s traumatic.” (ID 117, 52-year-old daughter of 73-year-old patient)

Another shared she was not acknowledged by the medical team on several occasions while physically present in her husband’s room, which negatively impacted trust in decision making:

“There were times that… a few [doctors] came in and looked at him and this and that and went back out and didn’t even ask who I was or acknowledge me period…If you can’t do that, how can I trust you to take care of the person that I love the most?” (ID 227, 63-year-old wife of 74-year-old patient)

Several Black family members shared that the ICU team limited their communication to asking for procedural consent. For example, this family member only spoke to the ICU team when they needed consent for surgery, even though her husband had been in the ICU for two months:

“They was calling to get permission to do that [left ventricular assist device] but I think [the doctor] could have communicated more with me than he did… Nobody never said nothing to me. I was worried about that [whether her husband would survive] myself. But I didn’t know who to talk to about it, so I just felt alone.” (ID 230, 68-year-old wife of 77-year-old patient)

In contrast, a White family member described strong rapport with the medical team, even supplanting traditional power dynamics that include addressing doctors with professional titles:

“I think we formed a relationship more than just doctor-patient… They would call me by first name. I, in turn, did the same for them. So they kind of removed that stale institutionalized hospital environment and made it more and brought it down to more of a personal level.” (ID 109, 66-year-old husband of 67-year-old patient)

### Perceiving tension between trusting clinicians’ competence versus intentions

Black family members’ trust in the medical team fluctuated based upon the clinician with whom they were interacting. In addition, some family members distinguished between trusting the technical expertise of clinicians but not necessarily their intentions, while others expressed having no choice but to trust the team caring for their loved one:

“Would I trust them? It is a tough question. I do. I would say I do because of the training that they have…But I feel like they would give up on you too quick.” (ID 121, 53-year-old wife of 56-year-old patient)

“We had no choice but trust, you know, I mean I was being very trusting to begin with because I was relying on them to do what they’re supposed to do.” (ID 219, 60-year-old sister of 63-year-old patient)

However, for many White family members, trust in the team was durable and founded in the team’s actions to build rapport with them:

“I felt like they got to know [patient] and got to know me and, and I felt like I could trust their thoughts.” (ID 342, 45-year-old wife of 48-year-old patient)

## DISCUSSION

In this qualitative study, Black and White family members of critically ill patients described their experiences of ddecision-making about life-sustaining treatments.

Both Black and White family members highlighted clinician transparency and personalized attention to patients as facilitators of effective SDM. However, their narratives also identified several opportunities for clinicians to improve SDM with Black families, such as supporting decisions made by families and providing time (when possible) to make decisions, recognizing families as patient experts and welcoming their advocacy, and demonstrating trustworthiness not only through technical expertise but also personalized and empathic care.

Black family members experienced substantial pressure surrounding decision making, which they reported paradoxically hindered their ability to make timely and well-considered decisions. Notably, Black family members experienced pressured decision making despite their loved ones having survivable critical illnesses in most cases and more commonly than loved ones of White family members in our sample. Structured approaches to SDM that reduce unnecessary pressure on families are needed. Time-limited trials, wherein families and ICU teams collaborate to define a duration and desired outcome of a trial of life-sustaining treatment, may be one such approach.^30–32^ Time-limited trials, when clinically appropriate, can protect families from the urgency and judgment surrounding decision making by establishing mutually agreed timelines and clinical metrics to determine whether life-sustaining treatments are achieving their desired benefit. It is critical that time-limited trials be grounded in collaboration between families and ICU teams, or else they may risk being paternalistic or even coercive.^33^

It is notable that three of the four themes identified in family interviews were not explicitly about decision making itself.^17,25,26^ Rather, code-switching, dismissal, and inconsistent trustworthiness may have indirectly limited opportunities for Black family members to ask questions, advocate for their loved ones, and ultimately, partner with the ICU team towards shared decisions. The root cause of all three is likely to be intergroup bias—that is, Black family members are in the out-group or socially dissimilar to the majority White ICU clinician workforce and thus may experience fewer automatic prosocial behaviors from clinicians and greater pressure to conform to in-group norms.^34^

Together, these three themes reinforce that information exchange and deliberation, identified in many conceptual models as the core of SDM, are neither sufficient for truly *shared* decision making nor viewed by minoritized families as the most important components of SDM.^1^ Rather, as has been advocated in prior work,^26^ it is crucial that clinicians first set the stage for SDM with all family members, but particularly minoritized family members, by empowering families as valued patient experts and demonstrating why the medical team can be trusted. Durable trust is the foundation for SDM—family members emphasized that it is not unidimensional or static, and it is a prerequisite for them to effectively partner in decision making. Trust can be challenging to build in an ICU setting, where medical teams are large, dynamic, and lack established relationships with families. This obstacle is further complicated by the reality that many Black families experience microaggressions that shape their perceptions and expectations for interactions with clinicians.^25^ Thus, it is crucial that all team members consistently demonstrate multiple dimensions of trust, including competence, empathy, and honesty.^35^ Research tools that measure SDM and education programs for SDM should also incorporate the central importance of power and trust, especially in similar clinical settings where intergroup bias is magnified.^36^

Black and White family members quantitatively reported a similarly high quality of clinician communication (Table 1); however, their qualitative narratives reflected disproportionately negative experiences for Black family members. The discrepancy may be due to ceiling effects, a common limitation of survey data methods.^37^ There are at least two possible explanations for the ceiling effect of the communication measure. First, social desirability bias may prevent patients and families from being critical of the medical team when responding to single-item survey measures.^38^ By contrast, in-depth qualitative interviews may provide permission and space to reflect on aspects of communication that could have been better. Second, Black families may have differing expectations for medical care compared to White families due to past negative experiences, which could explain why their quantitative assessments of communication quality were high but their qualitative experiences were strikingly worse.^39,40^ Interestingly, the survey measure inquiring about conflict with clinicians had more variation, in keeping with the qualitative data, suggesting it may be a promising outcome measure for future studies of inequities in clinical communication. Taken together, these observations highlight the importance of qualitative research when investigating health inequities.

A critique of these results may be that they only represent the perspective of family members on a bidirectional process between families and clinicians. We offer three responses. First, clinician perspectives on SDM have been codified through their authorship of clinical practice guidelines^1,41,42^; however, the priorities and needs of patients and family members, particularly those from structurally marginalized groups, have been infrequently centered.^43^ Second, the perspectives of families in this study’s sample are corroborated by our earlier work, in which we analyzed real-world decision-making conversations between Black and White families and ICU clinicians.^16^ In that prior work, we observed the same clinician behaviors endorsed by Black families in the current sample: clinicians shared limited medical information unless family members presented as highly health literate or socially connected, supporting the need for the code-switching described here; clinicians commonly dismissed Black family members’ emotional needs; and clinicians preferred limiting life-sustaining treatments rather than supporting families’ decisions when families preferred restorative care. Third, although clinician-family relationships are bidirectional, the medical team possesses greater power due to their specialized knowledge, skills, and decision-making role. As such, the onus is upon ICU team members to ensure that families have equitable, high-quality experiences with critical care and communication.

Strengths of this study include a majority Black sample to center the experience of Black families, a larger sample than is typical in qualitative research to enable robust racial comparisons, and corroboration by previous qualitative research on the same phenomenon.^16,25^ This study also has several limitations. First, these data were collected at a single institution in the Southern United States, where the function of race in communication may differ from other regions of the country. Although anti-Black racism is prevalent in all regions of the country^44^, additional studies from diverse geographies will be valuable. Second, family members may have been aware that the interviewer, in most cases, was a critical care physician, which may have limited what they shared about other critical care clinicians. However, family members expressed many negative sentiments in interviews, and if reporting bias were still present, the magnitude of racial differences in SDM experiences would only be larger than summarized here. Third, the demographics of the clinicians involved in these cases were unknown; thus, it was not possible to analyze the influence of identity concordance on families’ experiences. However, most critical care physicians are White and male, suggesting a high likelihood of identity discordance between families in this sample and physicians.^45,46^ Fourth, although coders were blinded to race, differences in language and grammar may have prevented effective blinding. Since editing transcripts for grammar is considered unethical^47^, we addressed bias related to potential unblinding by double coding and seeking consensus among three investigators for theme development. Further, we note that differences in language in the Southeastern United States are not only correlated with race but also with socioeconomic status and community of origin.

## CONCLUSION

In summary, Black family members of critically ill patients reported barriers to shared decision making with ICU medical teams that included experiencing pressure to make decisions, needing to code-switch by modifying communication and behavior to fit in, being disregarded by the medical team, and perceiving inconsistencies between multiple dimensions and levels of trust. These may be promising focus areas for future conceptual models and interventions focused on improving equity in ICU-based SDM.

## Supplementary Information

Below is the link to the electronic supplementary material.

ESM1 (DOCX 23.5 KB)

## Data Availability

Data will be made available after institutional board review and approval of a data use agreement.
