# Supplementary material for: Perspectives of Black and White Family Members on Medical Decision Making for ICU Patients
Source: J Gen Intern Med. 2026 Jan 29;41(7):1814–23. doi: 10.1007/s11606-026-10213-z (PMC13176412; doi:10.1007/s11606-026-10213-z)
Supplement: Supplementary file 1 — (DOCX 23.5 KB) [file 11606_2026_10213_MOESM1_ESM.docx]

**Supplement 1: COREQ checklist (submitted separately)**

**Supplement 2: Interview Guide**

1. How do you know [patient name]?
2. Why was he/she in the ICU?
3. Families are often asked to participate in making medical decisions in the ICU, including deciding whether their loved one will receive certain treatments like breathing machines, dialysis, or surgeries, or when to stop these treatments. What were the major decisions that you had to make in the ICU? How did you decide which treatments [patient name] should receive or not receive?
   1. Where did you get the information that you needed to make the decision? What other information would have been helpful to you?
   2. What did the doctors say or do that was helpful in deciding what to do for your loved one? What else would have been helpful? What things did they do or say that were not helpful or made things more difficult?
   3. How comfortable did you feel asking questions? What made you feel that way?
   4. How did other family or friends help you with the decision? Make it more difficult?
4. Did the medical team question any decisions that you made for your loved one? [If yes] What made you feel that way?
   1. How important is it for the medical team to support your decision or reassure you that you’re doing the correct thing?
5. Making medical decisions can be emotionally difficult. How did you feel when you were making decisions for your loved one in the ICU?
6. How did the ICU team help you cope? What else could they have done to help you?
7. In what ways did the ICU team perhaps make things worse or more difficult? What could they have done differently?
8. Many families talk about the importance of maintaining hope or faith when their loved one is seriously ill. Was this important for you? How did you communicate your hope to the team?
9. Hope is important for families, yet doctors often have to share bad news when patients are seriously ill. What advice would you give to doctors about doing both – being honest and allowing families to hold onto hope?
10. Most families and doctors agree that it is important to know and trust each other before engaging in shared decision making. This can be challenging in an ICU, where you may be making complex decisions with a doctor you have just met. What advice would you give to doctors about establishing trust and rapport in a short amount of time?
    1. If you can recall a doctor who did this well, what did they do?
    2. If you can recall a doctor who did not do this well or that caused you to distrust the team, tell me about that. What could the doctor/team have done differently?
11. Thinking back to your experiences in the ICU and making decisions for your loved one, how else could you have been better included or supported by the clinician(s) during the decision-making process? What advice would you give the medical team to support family members like you when they’re making complex decisions?

**Supplement 3: Codebook**

| **Code** | **Definition** |
| --- | --- |
| 1. CAREGIVER CHARACTERISTICS | Characteristics of the caregiver that are relevant to decision making |
| 1. Prior healthcare experience | The caregiver has professional healthcare experience or prior caregiving experience |
| 1. External responsibilities | Caregiver responsibilities external to the ICU that affected decision-making experience |
| 1. Role | Caregiver roles or perceptions of themselves that were relevant to ICU decision making |
| 1. Relationship to patient | Caregiver is the mother, father, daughter, son, etc. of the patient |
| 1. Other | Any caregiver characteristics not captured above |
| 1. ILLNESS | Circumstances specific to the medical condition of the patient that affected decision making |
| 1. Illness time course | Mention of ICU stay duration or chronicity of illness prior to ICU admission |
| 1. Medical circumstance | Any discussion of patient’s illness and prognosis of the patient’s illness while in the ICU |
| 1. DECISIONS MADE | The type of medical decision the caregiver was required to make affected the decision-making process |
| 1. Withdrawal or continuation of life support | There was a decision related to choosing either a measure that would prolong life (e.g., tracheostomy) or a transition towards comfort-based care |
| 1. Other |  |
| 1. SOURCES OF INFORMATION |  |
| 1. Medical team | The caregiver received medical information directly from the clinical team |
| 1. Social network | The caregiver received medical information from friends, coworkers, or other family members |
| 1. Other | Sources not captured above |
| 1. CARGEIVER PRESENCE | Frequency and manner in which the caregiver was able to check in on the patient |
| 1. Physical presence | Caregiver was often physically present in the ICU |
| 1. Other | Caregiver was unable to be physically present in the ICU, though maintained presence via phone call or other methods. |
| 1. POSITIVE CLINICIAN COMMUNICATION | Communication by the clinical team (physician, nurse, social worker, etc.) that the *caregiver perceived as effective or helpful* |
| 1. Partnership | The clinician included the caregiver as a valued member of the team |
| 1. Validation/reassurance | The clinician communicated to the caregiver that they approve of decision, personal quality (religious belief), etc. |
| 1. Other | Other not captured above |
| 1. NEGATIVE CLINICIAN COMMUNICATION | Communication by the clinician team that the *caregiver perceived as ineffective or unhelpful* |
| 1. Technical language | The clinician used language that was complex and difficult for the caregiver to understand |
| 1. Dismissal | Clinician behaviors that dismissed or devalued caregiver input |
| 1. Other | Other not captured above |
| 1. SUPPORTIVE CLINICIAN BEHAVIORS | Actions by the clinical team (physician, nurse, social worker, etc.) to support the caregiver |
| 1. Care for caregiver | Clinical team was attentive to the physical, emotional, etc needs of the caregiver |
| 1. Other |  |
| 1. VENUE FOR COMMUNICATION | The setting for communication about patient status or decision making |
| 1. Family meeting | Caregiver received information about patient status at planned meetings with the specific goal of updating the family |
| 1. Team rounds | Caregiver learned about patient status during clinical rounds |
| 1. Other |  |
| 1. POSITIVE EMOTIONS/SENTIMENTS | Caregiver emotions that carry positive valence, includes feeling validated, like they had authority/autonomy, etc. |
| 1. NEGATIVE EMOTIONS/SENTIMENTS | Caregiver emotions that carry negative valence, includes feeling dismissed, pressured, etc. |
| 1. RELIGION/SPIRITUALITY | The caregiver says that their religion or spirituality guided them on what decision to make |
| 1. HOPE | Code when interviewer prompts caregiver to discuss hope |
| 1. TRUST | Code when interviewer prompts caregiver to discuss trust; can be discussion of behaviors that elicited OR deteriorated trust |
| 1. KNOWLEDGE OF PATIENT PREFERENCES |  |
| 1. Present | Caregiver was aware of patient preferences for healthcare |
| 1. Absent | Caregiver was unaware of patient preferences for healthcare |
| 1. Conflicted | Caregiver did not agree with patient preferences for healthcare |
| 1. ADVOCACY | Caregiver effectively lobbied for self or the patient’s desires/opinions. Does *not* include simply feeling comfortable asking questions (that would be positive emotion) |
| 1. FAMILY CONFLICT | Caregiver disagreed or clashed with family member or other decision maker |
| 1. REPORTING | Caregiver reported negative experiences to administrative or clinical staff |
| 1. SUGGESTIONS | The caregiver offers suggestions about changes they would like to see; does not have to be related to clinician behavior |
